# Tsukamurella pulmonis Isolated in Sputum Culture During Workup for Tuberculosis: Colonization or Infection?

**DOI:** 10.7759/cureus.86438

**Published:** 2025-06-20

**Authors:** Navin Bhatt, Shital Khanal, Maurice Policar

**Affiliations:** 1 Internal Medicine, NYC Health + Hospitals/Elmhurst, Icahn School of Medicine at Mount Sinai, New York, USA; 2 Dermatology, B.P. Koirala Institute of Health Sciences, Dharan, NPL; 3 Infectious Diseases, NYC Health + Hospitals/Elmhurst, Icahn School of Medicine at Mount Sinai, New York, USA

**Keywords:** colonization, respiratory infection, sputum culture, tsukamurella, tuberculosis

## Abstract

*Tsukamurella pulmonis* is a rare, opportunistic actinomycete most often associated with respiratory infections in immunocompromised patients. We report the case of a young immunocompetent man with a remote history of completely treated pulmonary tuberculosis, in whom *T. pulmonis* was repeatedly isolated from sputum during routine screening. Despite these findings, the patient remained asymptomatic, with normal inflammatory markers and stable chest imaging, and therefore did not receive antimicrobial therapy. This case underscores the importance of correlating microbiological results with clinical and radiological assessments. It highlights that, in immunocompetent individuals, the presence of *T. pulmonis* in respiratory cultures may represent colonization rather than true infection, and unnecessary antimicrobial treatment can be avoided with thorough evaluation.

## Introduction

*Tsukamurella* species are aerobic, gram-positive, partially acid-fast actinomycetes widely distributed in environmental sources such as soil and water, but only infrequently encountered in clinical settings [1]. Over the past two decades, advances in molecular diagnostics have led to an increased recognition of *Tsukamurella* as an emerging opportunistic pathogen in humans [2]. The genus is most often implicated in catheter-related bloodstream infections, peritonitis in patients undergoing peritoneal dialysis, and rarely, cutaneous or pulmonary infections [1,3]. Most clinical cases of *Tsukamurella* infection are reported in immunocompromised individuals or those with underlying chronic illnesses, including malignancy, diabetes, or long-term corticosteroid use [4-8].

Among the various species, *Tsukamurella pulmonis* is notable for its potential to cause pulmonary disease, though such cases remain rare and are often associated with structural lung pathology or immunosuppression [9]. Pulmonary *Tsukamurella *infections frequently present with clinical and radiological features that can mimic tuberculosis or non-tuberculous mycobacterial disease, complicating initial diagnosis [10,11].

Yet, the significance of isolating *Tsukamurella *from respiratory specimens in immunocompetent patients is uncertain because colonization rather than true infection may occur [12,13]. This distinction is clinically important, as inappropriate treatment may contribute to antimicrobial resistance and unnecessary toxicity. Here, we report a case of *T. pulmonis* isolated from sputum in an immunocompetent patient with a stable clinical course, highlighting the diagnostic and therapeutic challenges posed by this rare finding.

## Case presentation

A man in his late 30s, with a remote history of completely treated pulmonary tuberculosis, was referred to the infectious diseases clinic following a positive acid-fast bacillus (AFB) culture result obtained during a routine pre-employment health screening. The patient was born in China and had immigrated to the United States 18 years prior. Before emigration, he had completed more than one year of anti-tuberculous therapy, initially receiving a four-drug regimen, which was subsequently narrowed to two drugs, resulting in complete resolution of his illness.

At the time of the referral, the patient reported no respiratory or constitutional symptoms. He denied cough, hemoptysis, sputum production, fever, or chills. He also had no complaints of chest pain, dyspnea, night sweats, or fatigue. The only notable symptom in the preceding period was transient weight loss during the COVID-19 pandemic surge, which he attributed to changes in diet and physical activity rather than any illness. He had no history of recurrent upper respiratory tract infections or previous treatment for bronchitis.

Physical examination was unremarkable, with normal vital signs and no abnormal findings on chest auscultation or inspection. Laboratory tests, including complete blood count, basic metabolic panel, and liver function studies, were all within normal limits. Notably, his C-reactive protein (CRP) was normal, and an HIV test was non-reactive.

**Figure 1:**
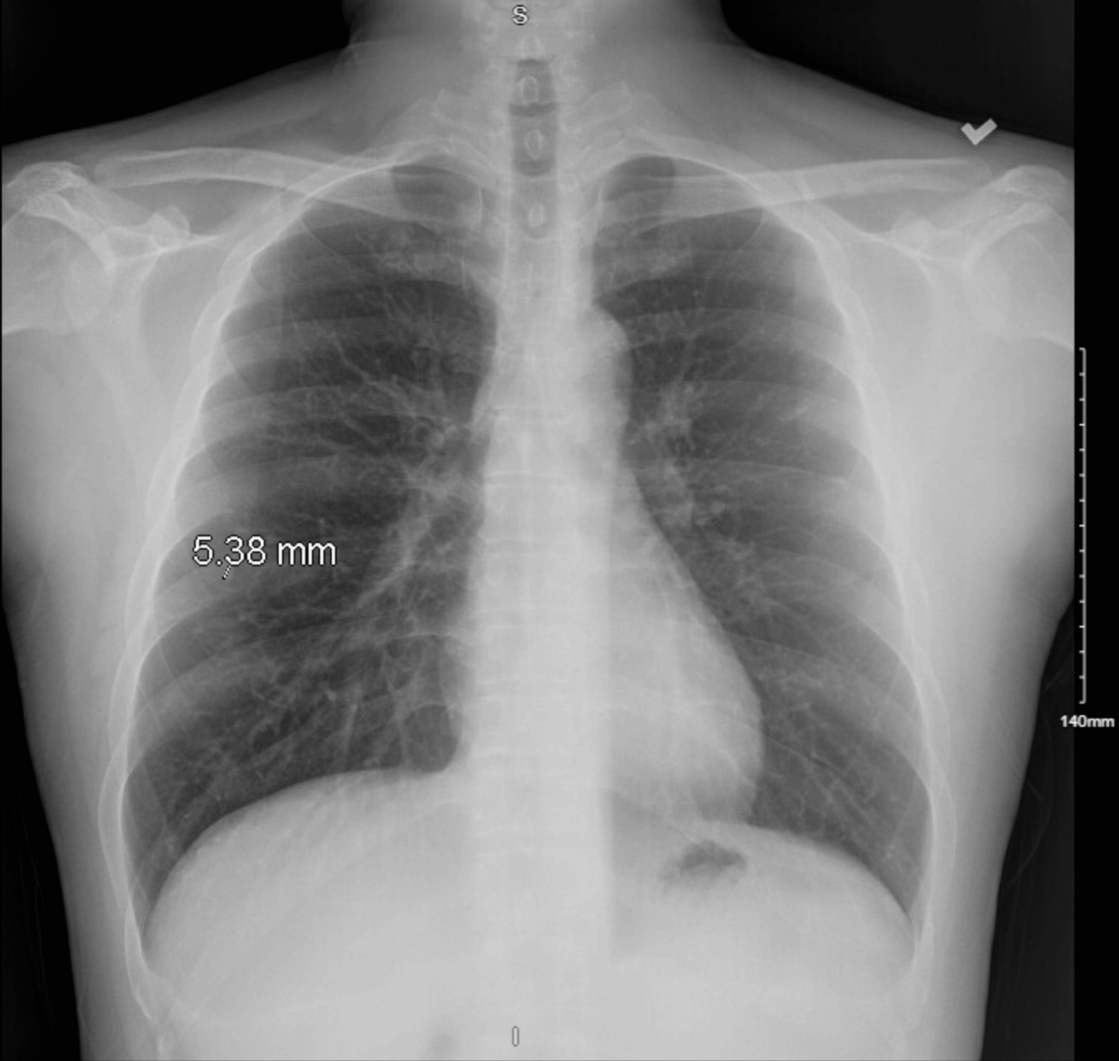
Initial chest X-ray demonstrating a 5.38 mm nodular density projected over the posterior aspect of the right 8th rib. While this may represent a vessel seen end-on, the size is slightly prominent for the location, and a small pulmonary nodule cannot be excluded.

**Figure 2:**
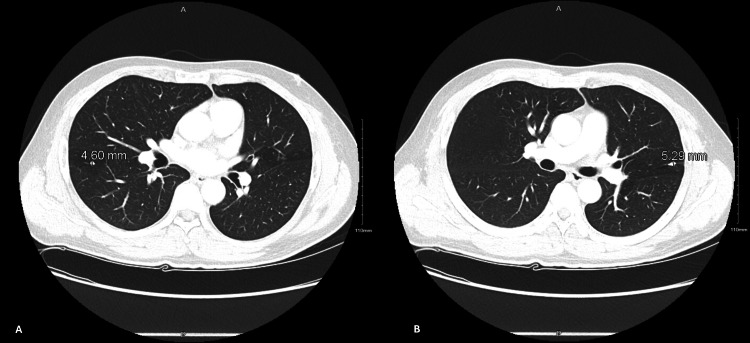
Contrast-enhanced chest CT demonstrating a 4.60 mm calcified nodule in the superior segment of the right upper lobe (A) and a 5.29 mm calcified granuloma in the lingular segment of the left upper lobe (B), both consistent with healed granulomatous infection.

The initial chest radiograph showed a 5.4 mm nodular density over the posterior right eighth rib, and a small pulmonary nodule could not be excluded (Figure 1). Subsequent contrast-enhanced chest computed tomography (CT) identified multiple calcified granulomas consistent with prior granulomatous disease, such as old tuberculosis, with no interval change compared to a study performed three years earlier (Figure 2). Additionally, no new infiltrates, masses, or cavitary lesions were observed.

Sputum smears for AFB were negative across all samples using fluorochrome staining. However, Gram staining of the sputum cultures revealed rare, branching Gram-positive rods. Two of three initial sputum cultures grew *T. pulmonis* in Mycobacteria Growth Indicator Tube (MGIT) liquid culture media, identified by matrix-assisted laser desorption ionization-time of flight mass spectrometry (MALDI-TOF MS). This unexpected finding raised concern for a possible *T. pulmonis* infection in an immunocompetent host.

Given the absence of symptoms, normal inflammatory markers, and stable imaging, the decision was made not to initiate antimicrobial therapy. For further evaluation, repeat sputum AFB cultures were obtained two months after the initial samples. Of the two new specimens collected, one again grew *T. pulmonis*. Throughout this period and on follow-up assessments at 21 and 36 months after initial presentation, the patient remained entirely asymptomatic, with no respiratory complaints or evidence of infection. He declined a repeat chest CT at one-year follow-up, citing the absence of symptoms and concern about unnecessary imaging. However, a chest X-ray performed two years later at an outside hospital showed stable calcified granulomas with no new changes. Over 36 months of follow-up, he experienced no respiratory tract infections and required no medical intervention related to his pulmonary status.

## Discussion

*T. pulmonis*, though rare, is increasingly recognized as a potential cause of pulmonary infections, particularly in patients with compromised immunity [1,8]. The genus shares phenotypic and staining similarities with other actinomycetes, such as *Nocardia *and non-tubercular mycobacteria, which can complicate both laboratory identification and the initial diagnostic approach [9]. To date, 17 species have been identified within the *Tsukamurella* genus, of which 12 have been implicated in various human infections [9].

Clinically, pulmonary *Tsukamurella* infections often mimic mycobacterial or non-tubercular mycobacterial infections, presenting with cough, fever, weight loss, fatigue, and sometimes hemoptysis [1,9,14]. Radiologically, they can show upper lobe infiltrates, consolidation, nodules, or cavitary lesions, making them easily mistaken for tuberculosis [14]. These similarities often result in misdiagnosis and inappropriate therapy until MALDI-TOF MS or advanced molecular methods, such as 16S ribosomal RNA and DNA sequencing, clarify the etiology [2,9].

*Tsukamurella* has been linked to a broad range of clinical syndromes beyond pulmonary infection, including catheter-related bloodstream infections, peritonitis, keratitis, skin and soft tissue infections, and device-associated illness [3,9]. While often dismissed as a contaminant, its pathogenic potential is supported when isolated repeatedly, especially in the appropriate clinical setting [8].

In our case, the patient had a remote history of tuberculosis but was otherwise immunocompetent and asymptomatic, with normal inflammatory markers and stable imaging over three years. *T. pulmonis* was repeatedly isolated from sputum by MALDI-TOF MS, but there was no evidence of clinical or radiological progression. This scenario highlights the crucial distinction between colonization, where the organism is present without tissue invasion or host response, and true infection [15,16]. Our findings are consistent with published reports that support the possibility of *Tsukamurella* acting as a commensal or transient colonizer, particularly in individuals with prior lung disease, and not necessarily as a pathogen in every instance [1].

Distinguishing colonization from infection is of real clinical importance. Unnecessary antimicrobial treatment exposes patients to adverse effects and fosters resistance [1,17]. In immunocompetent individuals without evidence of active infection, observation and regular follow-up are appropriate, as demonstrated by our patient’s benign course over three years. When true infection is suspected or confirmed, there are no standardized guidelines for antimicrobial therapy [1,10,18]. Case reports indicate that *Tsukamurella* species are typically resistant to penicillins and cephalosporins but susceptible to aminoglycosides, fluoroquinolones, macrolides, carbapenems, and sulfamethoxazole [1,19]. Cavitary pneumonia with *Tsukamurella* has been treated with rifabutin and fluoroquinolones for 3 to 6 months [4,6]. However, given the lack of robust evidence, therapy should be individualized and guided by susceptibility testing and clinical response.

## Conclusions

This case highlights the importance of a comprehensive, patient-centered approach when interpreting the isolation of rare organisms like *T. pulmonis* from respiratory specimens. In immunocompetent individuals without symptoms or evidence of active disease, such findings should prompt careful consideration of colonization rather than automatic assumption of infection. The distinction between colonization and infection can prevent unnecessary antimicrobial use, reducing the risks of resistance and adverse effects. Clinicians must integrate clinical presentation, imaging, and laboratory data to guide management decisions, especially in the absence of established guidelines. Ongoing vigilance and further research are essential to refine our understanding of the clinical significance of *T. pulmonis* in diverse patient populations.
